# Piecewise multivariate modelling of sequential metabolic profiling data

**DOI:** 10.1186/1471-2105-9-105

**Published:** 2008-02-19

**Authors:** Mattias Rantalainen, Olivier Cloarec, Timothy MD Ebbels, Torbjörn Lundstedt, Jeremy K Nicholson, Elaine Holmes, Johan Trygg

**Affiliations:** 1 Department of Biomolecular Medicine, Division of Surgery, Oncology, Reproductive Biology and Anaesthetics (SORA), Faculty of Medicine, Imperial College, London, SW7 2AZ, UK; 2 Research Group for Chemometrics, Institute of Chemistry, Umeå University, Umeå, S-901 87, Sweden; 3 Department of Pharmaceutical Chemistry, Uppsala University, Sweden; 4 AcurePharma, Uppsala, Sweden

## Abstract

**Background:**

Modelling the time-related behaviour of biological systems is essential for understanding their dynamic responses to perturbations. In metabolic profiling studies, the sampling rate and number of sampling points are often restricted due to experimental and biological constraints.

**Results:**

A supervised multivariate modelling approach with the objective to model the time-related variation in the data for short and sparsely sampled time-series is described. A set of piecewise Orthogonal Projections to Latent Structures (OPLS) models are estimated, describing changes between successive time points. The individual OPLS models are linear, but the piecewise combination of several models accommodates modelling and prediction of changes which are non-linear with respect to the time course. We demonstrate the method on both simulated and metabolic profiling data, illustrating how time related changes are successfully modelled and predicted.

**Conclusion:**

The proposed method is effective for modelling and prediction of short and multivariate time series data. A key advantage of the method is model transparency, allowing easy interpretation of time-related variation in the data. The method provides a competitive complement to commonly applied multivariate methods such as OPLS and Principal Component Analysis (PCA) for modelling and analysis of short time-series data.

## Background

Metabolic profiling, (also referred to as metabonomics [1] or metabolomics [2]) is a rapidly developing field in which the levels of hundreds to thousands of low molecular weight metabolites are simultaneously profiled in biofluids, cells and tissues. The methodology is well-established for characterizing disease states, toxicity and differences in physiological condition [1,3-10] and for extracting metabolite patterns associated with these conditions. In many experiments the biological system is followed over time, generating a multivariate metabolic time course. For example, staging of a disease process may be more important than merely determining its presence or absence. The ability to accurately define and predict disease stage also has obvious application in assessment of response to therapeutic intervention. Ideally such time-series data should be well sampled in the time domain and have an adequate statistical experimental design, which is an essential component for the outcome of the study and quality of data [11]. However, for practical reasons collection of an optimal dataset is not always possible.

In the case of multivariate time-series data with low sample rate, many classical statistical methods are not appropriate for analysis and characterization of the time related variance due to the low number of time-points and in some cases inability to handle multivariate data. Principal Component Analysis (PCA) [12] has previously been applied for the analysis of time-series data in metabonomics [13-17], allowing visualization of the main time-related patterns of variation. PCA has the objective of describing the main variance in the data in a low-dimensional subspace spanned by a few linear components. Since PCA does not explicitly model time-related variation, it does not provide an optimal representation of time-related data. In addition, the PCA model usually has more than one PCA component, making subtle changes described by multiple components hard to interpret. Partial Least Squares (PLS) regression [18] with the time as regressand has also been used for analysis of time-series metabonomic data [19,14,15,20], but the assumption of a linear relation between descriptor variables and time is valid only under some specific circumstances, but not in the general case. The PARAFAC model [21,22] provides a generalisation of PCA to data matrices of higher dimension. In this case, the three-way structure of the data consists of [Animal × Time × Variables]. The reason why 3-way methods are not used in this study is because the NMR spectral profile (over all animals) do not preserve neither the rank, nor the spectral profile over all time points, which violates the 3-way method assumption of tri-linearity. Smilde et al. [23] described a generalization of the ANOVA approach to the multivariate case for data generated from an experimental design, labelled as ANOVA-Simultaneous Component Analysis (ASCA), with application to time-series data. However, in ASCA the time related effects are assumed to be linear in relation to time, which is rarely a valid assumption, neither does the ASCA method providing a predictive model. For short and univariate time-series, piecewise linear modelling methods can be used to describe progression over a time-series, which is similar in some aspects to the method proposed here for the multivariate case. Other statistical methods applied for the analysis and modelling of time-series data in omics biology include Clustering [24], Dynamic Bayesian Networks [25] and Batch Statistical Process Control [14,26,27]. Applications of time-series analysis have been described by Trygg and Lundstedt [11] in a review of chemometric techniques applied in metabonomics, and some of the current issues with regard to analysis of time-series gene expression data were reviewed by Bar-Joseph [28].

Here a new method for piecewise multivariate modelling of time-series spectroscopically generated metabolic data is proposed, which can be used for characterization and modelling of short (less than 20 time points) and sparsely sampled (sampling frequency is low relative the time-scale of the events studied) time-series data of high dimension. The method is well suited for analysis of spectral metabolite profiles where variables are intrinsically multicolinear, but is also generally applicable to other types of omics data. The suggested method also provides descriptive information, enables visualization and establishes a predictive model based on time-related variance, putting focus on effects seen between local time-points. The proposed method is based on multivariate piecewise models, where each sub-model describes changes occurring between neighbouring time points in a series of time frames over the time course, here the piecewise model is an Orthogonal Projections to Latent Structures [29] (OPLS) model. Overall, the set of sub-models describe the time-related changes over the full time also encompassing the modelling of non-linear changes in relation to time. Visualization of the piecewise multivariate model can be accomplished by investigation of sub-models separately, cumulatively over all time frames and as a time-trajectory. One can interpret the local changes as the rate of metabolic change in the time course. This aspect of explicitly investigating the multivariate characteristics of change, together with the magnitude of change over time in a biological system, has not been explored previously to the knowledge of the authors. In addition we show how this approach can be used for prediction of the time-point along a time-series, based upon measured metabolic profiles. Prediction of time-point by the model could be used for monitoring disease stage over time as well as for evaluation of the efficacy of an intervention, e.g. by assessing change in predicted disease stage after an intervention.

The paper is organized as follows. A brief introduction to the OPLS method is given, followed by a detailed description of then proposed piecewise multivariate modelling of sequential data. Finally the method is demonstrated on both simulated data and metabolic profiling data and results are compared to results from PCA analysis as well as linear OPLS regression modelling.

## Results

### Algorithm

With the objective of describing the time-related variance in the data, a set of multivariate piecewise models is estimated, describing the transitions between metabolic states in neighbouring time points, using the OPLS algorithm. Each model establishes a function for the transition between two time points will be called a *sub-model *in the following sections. A distinction is made between the piecewise approach, consisting of a set of OPLS sub-models, and the OPLS regression approach where the descriptor matrix is regressed against the time using all time points in a common model, thus, assuming a linear relationship between the data and time. Let the matrix **X **[*N *× *K*] (for *N *observations and *K *descriptor variables) represent the matrix of descriptor variables, where each observation, e.g. a metabolic profile, is a row-vector of **X**. The data vector for time-point *i *for individual *n *is denoted by **x**_n,i_. **Y **is the response matrix [*N *× *M*] (for *N *observations and *M *response variables). T represents the total number of time-points, resulting in T-1 sub-models. Throughout the paper matrices are represented by bold uppercase letter, vectors bold lower-case, scalars are represented as italics, *p*(·) represents a probability density and tr(·) is the trace function.

#### PLS and OPLS methods

Partial Least Squares regression (PLS) [18] has been used successfully for estimation of multivariate regression and discriminant models in many applications, especially in cases when descriptor variables are multicollinear and noisy, and when the number of variables exceeds the number of observations, which is common for e.g. spectroscopic and other omics data. For data with systematic variation, which is orthogonal to the regressand, the number of PLS components required for an optimal predictive model normally exceeds the rank of the **Y**-matrix. In such cases, the Orthogonal Projections to Latent Structures (OPLS) method [29], which has an integrated Orthogonal Signal Correction filter [30-33] specifically designed for PLS, will benefit the analysis. This allows the estimation of an optimal model (in the predictive sense) with a single predictive component for the single **Y**-variable case, contrary to the PLS model which may have several components if structured **Y**-orthogonal noise is present in data. This property of the OPLS algorithm, guaranteeing a single predictive component for the single **Y**-variable case, is utilized in the method described here. It confers an advantage compared to other similar multivariate projection methods, in terms of clearer interpretation of the model and enabling a straightforward extension to the piecewise model described here. The simplicity of interpretation is due to the separate modelling of correlated components and **Y**-orthogonal components in the OPLS model.

#### Estimation of piecewise sub-models

Estimation of a multivariate sub-model between time point *i *and *i+1 *can be treated as a discriminant analysis problem between two time points, describing the time (**Y**) as a function of the descriptor matrix (**X**). Let the **X**_i _[*N*_i _× *K*] matrix consist of training data from time *t *= *i *and *t *= *i+1 *with *N*_*i *_observations, and let the **Y**_i _[*N*_i _× 1] matrix to be a dummy matrix of zeros and ones, indicating which observation belongs to time point *t *= *i *and *t *= *i+1 *respectively.

The OPLS algorithm decomposes **X**_i _into a predictive weight vector, **w**_p,i _[*K *× 1], describing the direction in the *K*-dimensional space between the two time points (*i *and *i+1*), and a predictive score vector, **t**_p,i _[*N*_i _× 1], representing the orthogonal projection of **X **onto **w**_p,i _(Equation 1). If **Y**-orthogonal variance is present in the data, the optimal predictive PLS model would include more than one PLS component, which in the OPLS model is equivalent to the estimation of additional **Y**-orthogonal components in addition to the predictive component in the model (Equation 1). This results in the guarantee of a single predictive component **w**_**p,i**_, describing the discriminative (locally time related) direction in **X**_i_, and *A*_*o *_**Y**-orthogonal components with loading matrix **P**_o,i _[*K *× *A*_o_] and score matrix **T**_o,i _[*N*_i _× *A*_o_], describing the systematic **Y**-orthogonal variation present in the data, if any.

(1) **X**_i _= **t**_p,i _**w**^**T**^_p,i _+ **T**_o,i _**P**^**T**^_o,i _+ **E**_i_

The **Y**-orthogonal variance may be analyzed further, either separately or together with the **X **residuals (**E**_i_) to understand the variance patterns present in the data that are not time-related, which may provide information of systematic instrumentation errors or biological variation not directly related to time but which may still be of value.

In Equation 1, **w**_p,i _may be interpreted as the direction of change in the 'metabolic space' in the local time frame, describing the transition between two neighbouring time points, *i *and *i+1*. **w**_p,i _may also be interpreted as an approximation to the derivative of the time dependent function of the metabolic state. **w**_p,i _only describes a direction but does not contain any information quantifying the magnitude of the change in each time frame. An intuitive measurement of the magnitude of change would be the Euclidean norm of the predictive score vector ||**t**_p,i_||. However the Euclidean norm may be affected by even moderate outliers and is therefore not an optimal choice. Instead we use the median distance in the score space as the metric for the magnitude of change (Equation 2).

(2) *d*_i _= |median(**t**_*p*
,i_) - median(**t**_p,i+1_)|

(3) **w**_dist,i _= **w**_p,i _*d*_i_

**w**_dist,i _is then defined as **w**_p,i _weighted by a scalar (*d*_*i*_) defining the magnitude of the change in the local time frame *i *(Equation 3), thus incorporating the information about the direction as well as magnitude of change. **w**_dist _will be referred to as the magnitude weights and may be used as a way of describing and visualizing the profile of time-related change in any given sub-model. **w**_dist _is also comparable in magnitude between the different sub-models, contrary to **w**_p_, which is scaled to unit norm.

#### Interpretation of time related changes in model

By applying elementary vector algebra we also define the cumulative **w**_dist_, **w**_dist,cum_, which represents the total time related changes as described by the sub-models between *t *= 1 and *t *= T (Equation 4). This provides useful information for interpretation and visualization of the time related changes described by the sub-models over the whole time-series.

(4) **w**_*dist*,*cum*,*i *_= **w**_*dist*,*t*=1 _+ **w**_*dist*,*t*=2 _+ ... + **w**_*dist*,*t*=*i*_, *t *= 1...*i*

**w**_dist,cum _provides information on the overall change from a given reference point (e.g. t = 1). This enables us to not only track the changes in the local time frames, but also to depict the accumulated change over the time course. **w**_dist,cum _may prove to be useful for investigations of systems where there is a change occurring from a homeostatic state or when studying the recovery over time after a perturbation to establish whether the system returns either to the biological state prior to the perturbation, or alternatively, to a new state. A return back to the original biological state would in result in a **w**_dist.cum _vector close to a vector of zeros. For visualization purposes, and to summarize the changes described by **w**_dist.cum _vectors, PCA may be applied on the **W**_dist,cum _matrix to visualize the major patterns of time-related variation in a low dimensional subspace, describing the main changes in the time-series. The low dimensional representation of the time points provides an overview of the relationship and similarity between the temporal states, or stages, rather than maximizing the amount of modelled variation in the original data, hence providing a less noisy visualization of the time-related variation in the data compared to a conventional PCA trajectory.

#### Prediction of time point

Time predictions for new observations are carried out in two steps. First the sub-model that best fits the new observation is established and within this sub-model a more detailed time prediction is then made. The decision of which sub-mode fits best the test-set observation is determined by two likelihoods. The first is p_T2_(**t**_p,test_|m_i_), the likelihood for a test-set observation (represented by the predicted score, **t**_p,test_) to fit to the sub-model (with set of parameters m_i_), based on the score (**t**_p_), where the likelihood is based upon Hotellings T^2 ^statistic estimated from the training data. The second is based upon analysis of the model residual vector (**e**). Using the distribution of the residuals from the training set we can calculate the Q-statistics [34], or alternatively DmodX [35], which shows similar characteristics. The Q-statistic (Equation 5), is based upon the sums of squares of the residuals, which is used to estimate the likelihood p_Q_(**e**|m_i_) for the test-set observation based on the Q-statistic from the training data. Q-statistics for residual analysis were described by Jackson [36,37]. Equations 5–8 describe how the parameter *c*, which follows an approximate *N*(0,1) distribution, can be calculated [36,37], leading us to the calculation of P_Q_(**e**|m_i_) (Equation 9). In Equation 5 **e**_test _represents the residual vector for a test-set observation. In equation 6, Σ_**E***i *_is the covariance matrix of **E**_*i*_, which is the residual matrix for the training data for sub-model *i*. In equation 9 *c' *represents an instance of *c *as calculated in Equation 8 for a specific test-set observation.

(5) *Q*_*test *_= **e**_*test*_^**T**^**e**_*test*_

(6) $$\begin{array}{l} \theta_{i,1}=\text{tr(}\Sigma_{E_{i}})\\ \theta_{i,2}=\text{tr(}\Sigma_{E_{i}^{2}})\\ \theta_{i,3}=\text{tr(}\Sigma_{E_{i}^{3}}) \end{array}$$

(7) $$h_{i,0}=1-\frac{2\theta_{i,1}\theta_{i,3}}{3\theta_{i,2}^{2}}$$

(8) $$c=\theta_{i,1}\frac{{\left(\frac{Q_{test}}{\theta_{i,1}}\right)}^{h_{i,0}}-\frac{\theta_{i,2}h_{i,0}(h_{i,0}-1)}{\theta_{i,1}^{2}}-1}{\sqrt{2\theta_{i,2}h_{i,0}^{2}}}$$

(9) p_Q_(**e**_test_|m_i_) = p(*c*<=*c'*), *c*~N(0,1)

Here p_Q_(**e**|m_i_) and p_T2_(**t**_p_|m_i_) are treated as independent, which should be acceptable in most cases of application. The joint probability for the new observation to belong to a given sub-model is then calculated as described in Equation 10 and Equation 11.

(10) p(m_i_|**x**) = *z*^-1^p(m_i_) p_Q_(**e**|m_i_) p_T2_(**t**_p_|m_i_)

(11) $$z=\sum_{i=1}^{T-1}p(m_{i})p_{T2}(t_{p}|m_{i})p_{Q}(e|m_{i}),\;T\text{-1 = number of submodels}$$

Equation 10 provides selection criterion for selecting the sub-model with the best fit, which is used for the prediction (Equation 13). The prior probability of each sub-model, p(m_i_), in Equation 10 can be used to if there are known prior probabilities for each sub-model, or otherwise assigned uniformly for all sub-models. Time ($\hat{Y}$) is predicted as described in Equation 12 and 13, where **X**_pred.new _is the prediction set **X **matrix after **Y**-orthogonal variation has been removed, **X**_pred _is the prediction set matrix, **T**_pred.o _is the **Y**-orthogonal scores matrix calculated from **X**_pred_, **P**_training.o _represents the matrix of **Y**-orthogonal loadings derived from the training **X **matrix, **y**_offset,i _is equal to time point *i *and **B**_i _is the matrix of OPLS predictive coefficients for the selected sub-model *i*.

(12) $$X_{pred.new}=X_{pred}-T_{pred.o}P_{training.o}^{T}$$

(13) $$\hat{Y}=y_{offset,i}+X_{pred.new}B_{i}$$

### Testing

#### The simulated data set

**Figure 1:**
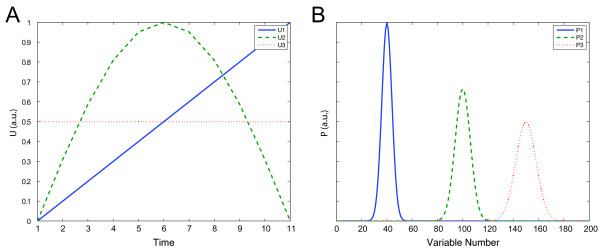
(**A**) The variation over time for the two time dependent latent variables in the simulated data set, and a variable independent of time. (**B**) Loading profile for the latent variables in the simulated data set.

To illustrate some of the properties of piecewise multivariate modelling approach a tractable example based on simulated data was used, which has both linear and non-linear time-related variation present. A spectral-like data set with 200 spectral variables, 11 time points, and 100 replicates for each time point was simulated using a bilinear model. The data contain two time dependent components, one non-linearly (**u**_1_) and one linearly (**u**_2_) related to time (Figure 1A) in addition to a constant component (**u**_3_) which contain only random variation, described in Equation 14. Each one of these three components is related to a specific spectral profile (**p**_1_, **p**_2_, **p**_3_) (Figure 1B). Random variation (ε ~ *N*(0,0.1)) was added to the time dependent latent variables for each time point and each observation.

(14) $$\begin{array}{l} \text{j}=\text{1,2}...\text{100 (Number of replicates)}\\ t=\text{[0,0}\text{.1,0}\text{.2}...\text{1}\text{.0]}\\ {\text{u}}_{\text{1,i,j}}=\text{sin(}\pi {\text{t}}_{\text{i}}\text{) + }\varepsilon \;\\ {\text{u}}_{\text{2,i,j}}={\text{t}}_{\text{i}}+\varepsilon \\ {\text{u}}_{\text{3,i,j}}=\text{0}\text{.5}+\varepsilon \\ x_{\text{ij}}={\text{u}}_{\text{1,i,j}}p_{\text{1}}^{\text{T}}+{\text{u}}_{\text{2,i,j}}p_{\text{2}}^{\text{T}}+{\text{u}}_{\text{3,i,j}}p_{\text{3}}^{\text{T}} \end{array}$$

#### PRINCIPAL COMPONENT ANALYSIS

**Figure 2:**
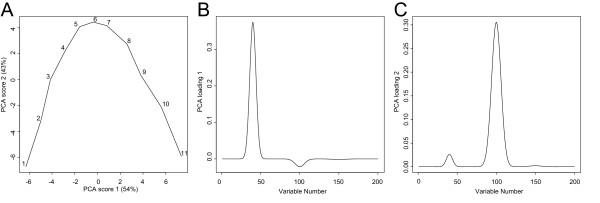
Simulated data set visualized as a PCA-trajectory. (**A**) PCA scores trajectory plot of component 1 and 2. (**B**) PCA loading component 1. (**C**) PCA loading component 2.

Results from PCA analysis of the simulated data is shown in Figure 2A–C visualized as a PCA trajectory plot, where the centroids in the score space are calculated for each time-point and then connect to form the trajectory (Figure 2A) (data was mean-centred prior to analysis). The loading plots for component one and two (Figure 2B and 2C) show that the two sources of variation in the simulated data are slightly confounded between the two PCA components.

#### OPLS REGRESSION

**Figure 3:**
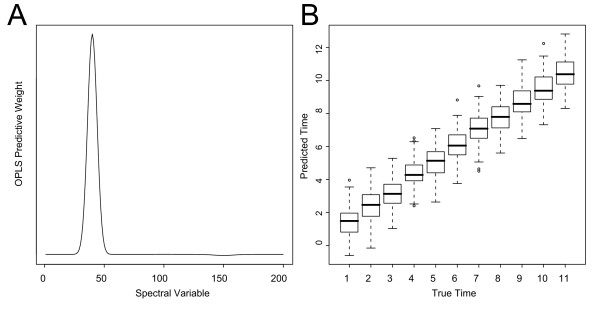
OPLS regression modelling of the simulated data set (linear regression against time). (**A**) OPLS predictive weights (**W**_p_), indicating the parts of the descriptor data that are modelled. (**B**) Time predictions results for the test-set plotted against the true time represented as a boxplot.

We investigate the same data set using the OPLS regression approach and regress the simulated spectral data against time, using one predictive component. Figure 3A shows OPLS predictive weights, indicating the predictive (**Y**-related) variation that is described by the OPLS model. As expected, only the leftmost peak in the spectral profile is given any weight in the model, while the time related, but non-linear component, is not present (compare with Figure 1). Predictions of a test-set (an independently drawn sample from the same distribution and with the same number of observations as the simulated training data set) using the OPLS model give a Root-Mean-Square Error of Prediction (RMSEP) of 9.7% (Figure 3B).

#### PIECEWISE MULTIVARIATE MODELLING

**Figure 4:**
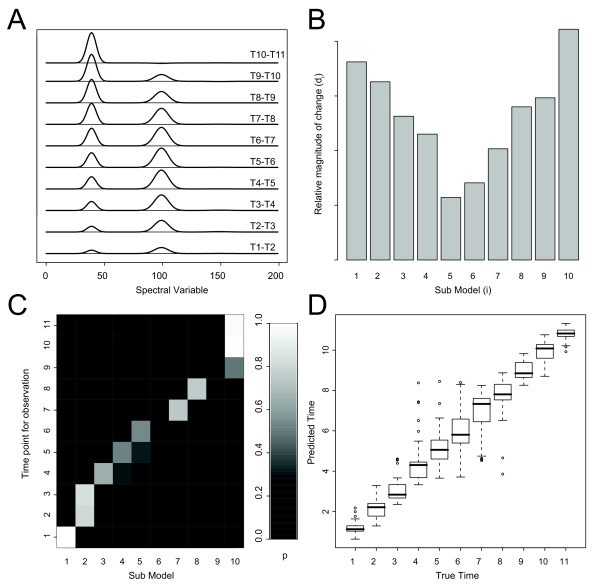
Modelling and prediction of the simulated data set. (**A**) Cumulative magnitude weights (**W**_dist.cum_) for each sub-model. (**B**) Distance between time points in each sub-model (*d*_i_) (**C**) Probabilities for one observation over all time points to belong to each sub-model (in prediction). (**D**) Predicted time for each observation in the test-set plotted against the true time represented as a boxplot.

In the piecewise model, the cumulative magnitude weights **W**_dist,cum _illustrate the cumulative change in the system over the time-series (Figure 4A). Figure 4B illustrates the relative magnitude of change (d_i_) in each sub-model, allowing the magnitude of change in each sub-model to be established. Deviations from the expected symmetrical magnitude of change profile (Figure 4B) for the simulated data set are a direct effect of the Gaussian noise present in the data set. It can be seen that d_i _approximately traces the gradient of the non-linear time-related component (Figure 1A) as expected. For the time-point predictions, the probability of sub-model membership was calculated for each of the test-set observations. The probabilities for all time points from one time-series is shown as an example in Figure 4C. The lack of symmetry seen in Figure 4C is because each sub-model (x-axis) represents a model between two neighbouring time-points. Therefore, an observation (y-axis) has the potential to fit fairly well into both of the adjacent sub-models, or one of them. The time predictions (RMSEP = 6.5%) based on the best fitting sub-model are displayed in Figure 4D in the form of a boxplot, indicating successful predictions. The larger prediction errors observed for time-points 4–8 (Figure 4D), compared to earlier and later time-points, are due to lower signal to noise level for these time-points. This is an effect of the time related component U_2 _(Figure 1A), which has a lower amount of time-related change over these time-points, while the noise level remains constant over the time course. The RMSEP is similar to the OPLS regression model, which is expected in this case since there is one latent variable in the simulated data that is linearly related to time. Crucially, the linear OPLS regression only models the linearly related time variation in the spectral profile, while the piecewise model shows both the linear and non-linear time-related variation in the data. This provides a model demonstrating a more complete representation of the time-related variation, enabling a more comprehensive interpretation.

#### The mercury II chloride data set

**Figure 5:**
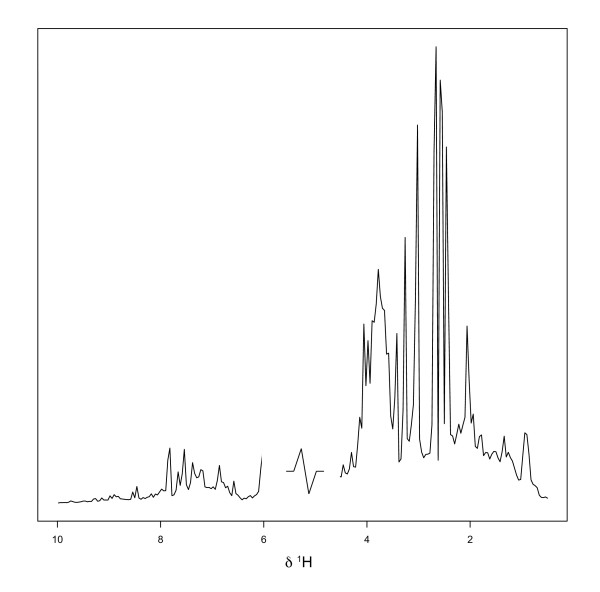
Typical integrated ^1^H NMR spectrum from the HgCl_2 _data set.

To test the method on real data, we used data from a renal toxicity study using mercury II chloride to induce a proximal tubular damage [38] in the rat. This is a ^1^H NMR based metabonomic study of rat urine with data from seven time points (pre-dose, 0 h, 8 h, 24 h, 48 h, 72 h, 96 h) and including ten animals in total. Prior to analysis the data were pre-processed using standard methods. First the spectra were interpolated to a common chemical shift scale using cubic spline interpolation. The region corresponding to water and urea resonances (δ 4.5 – 6) was excluded from each spectrum and the spectral intensity was subsequently integrated over adjacent δ 0.04 ppm width bins. Each spectrum was normalized to the total sum of 100 units to reduce the overall dilution effect due to inter animal variability in urine excretion rates. A typical integrated NMR spectrum after pre-processing is shown in Figure 5. After 48 hours five animals were sacrificed, rendering N = 5 animals to be left in the study after 48 h.

#### PRINCIPAL COMPONENT ANALYSIS

**Figure 6:**
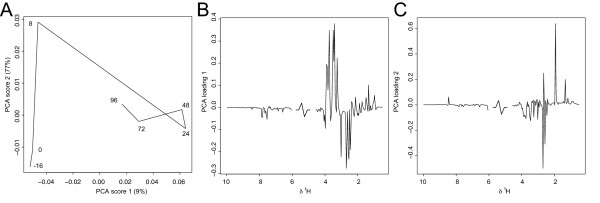
Mercury II chloride toxicity data visualized by PCA. (**A**) PCA score trajectory plot in component one and two. (**B**) PCA loading component 1. (**C**) PCA loading component 2.

Results from PCA analysis of the HgCl_2 _data is shown in Figure 6A–C visualized as a PCA trajectory plot, where the centroids in the score space are calculated for each time-point and then connect to form the trajectory (Figure 6A). Figure 6B and Figure 6C show the loadings for each one of the two PCA components calculated. In many instances the pattern of change between time points is a combination of variation in more than one PCA component, making it hard to interpret the unique pattern of change over different regions of the time-series, especially when these changes may be subtle.

#### OPLS REGRESSION

**Figure 7:**
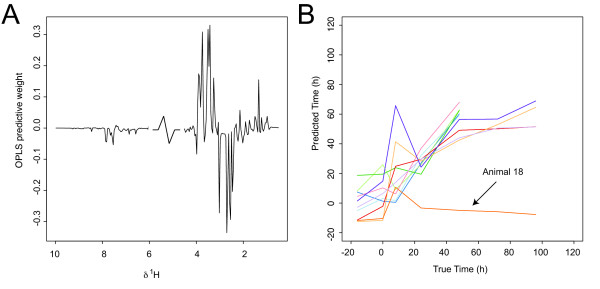
Mercury II chloride toxicity data modelled by OPLS regression against time. (**A**) OPLS predictive weights (**W**_p_) (**B**) Time after administration of HgCl_2 _plotted against the time predicted by the OPLS model. Results are shown from cross-validation where all time points from one animal at the time was left out from the modelling to provide the test-set.

The HgCl_2 _data set was analyzed by linear OPLS regression against the time using one predictive component and one time-orthogonal component (based on cross-validation). Interpretation of the predictive component from the OPLS regression model against the time provides information about variance linearly related to time (Figure 7A). The predictive performance of the model was evaluated by cross-validation, where all time-points from one animal at a time were left out from the model estimation and used as a test-set, to evaluate time-predictions (Figure 7B). The Root-Mean-Square Error of Cross-Validation (RMSECV) was 22.9%.

#### PIECEWISE MULTIVARIATE MODELLING

**Figure 8:**
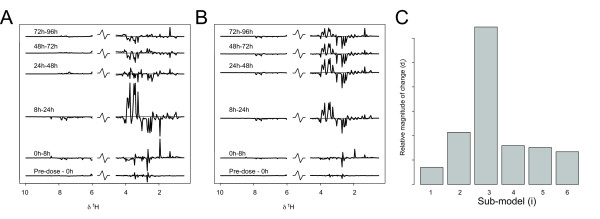
Mercury II chloride toxicity data set modelled by piecewise multivariate modelling. (**A**) Magnitude weights (**W**_dist_) for each sub-model, describing the differential time related changes in the NMR spectra. (**B**) Cumulative magnitude weights (**W**_dist.cum_), showing the accumulated changes over the time course. (**C**) Magnitude of change (d_i_) for each sub-model over the time-course.

Figure 8 shows the magnitude weights (**W**_dist_) (8A) and the cumulative magnitude weights (**W**_dist.cum_) (8B), describing the metabolic changes over the time-series. The magnitude weights (**W**_dist_) carry information about profile (direction) and magnitude of the changes in each local time frame. The cumulative magnitude weights (**W**_dist.cum_) represent the accumulated metabolic state. Inspection of the magnitude of change in each piecewise model provides further information about the degree of change at different parts of the time-course (Figure 8C). Here we can see that the largest magnitude of change is occurring in time frame three (8–24 h).

**Figure 9:**
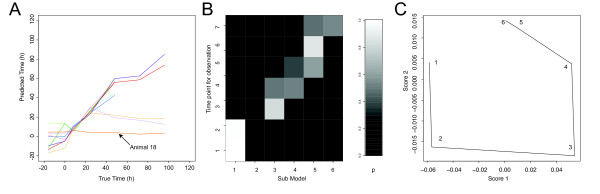
Mercury II chloride toxicity data modelled by piecewise multivariate modelling. (**A**) Predicted time by the piecewise multivariate model plotted against the true time after administration of HgCl_2_. Results are shown from cross-validation where all time points from one animal were left out from the modelling to provide the test-set are shown. Animal 18 (marked by arrow) is a non-responder, which is also indicated by the prediction results. (**B**) Probability of sub-model membership for each sample for one animal. (**C**) Visualization of **W**_dist.cum _as a trajectory in a low-dimensional space, estimated by PCA.

The predicted time based on cross-validation, as described in the previous section, plotted against the true time point for each animal in the study is shown in Figure 9A (RMSECV = 23.2%), showing a different pattern of prediction results compared to the OPLS time regression model (Figure 7B). We note that the predictions are less variable in the early time points compared to the OPLS regression approach. However, in the latter part of the time-course the OPLS regression model performs quite similarly to the piecewise approach, indicating that there is some variation in the metabolite profiling data linearly related to time. For the time points up to 48 h the predictions are relatively good, but as the observation decrease from n = 10 to n = 5 after 48 h, the model becomes less reliable as evidenced by the poorer predictions. However, important information can still be extracted, for example animal 18 (Figure 7B) is clearly a non-responder, which was confirmed by histopathology data, showing no renal damage, and clinical chemistry data, showing osmolality and glucose levels in the same range as control animals, indicating a negligible effect of the toxin on this animal. Figure 9B shows the probabilities for all time-points of animal 16 for fitting each sub-model, as an example of how sub-models are chosen.

To provide an overview of the time-related changes, PCA was used to visualize **W**_dist.cum _as a trajectory (Figure 9C). The main information provided by this plot is for assessment of whether the perturbed system has returned to its starting point, and to provide a visualization of the overall time-events. In Figure 9C we can see a trend towards the metabolite profile returning to a state close to that prior to the administration of HgCl_2_. However, over the study duration the recovery was not complete, which may either be due to presence of irreversible injury inflicted by the toxin or that the time span over which the study was carried out was too short to allow observation of a full recovery.

### Implementation

A R package [39]  implementing the method described is available upon request from the corresponding author. 

## Discussion

We have presented a framework for analysis of short time-series multivariate data, typical of post-genomic (omic) biology, using piecewise multivariate modelling and we demonstrated the method on metabolic profiling data. However, this approach is applicable to other types of omics data as well. The proposed method facilitates a transparent model allowing straightforward interpretation of time related variation in the data over the time course. Prediction of the time-point for a new sample is possible. The method has applications in areas such as monitoring and prediction of disease progression or the effects of a perturbation over time, allowing for evaluation of different types of interventions. The piecewise approach makes no assumption of linear relationship between the data and time and is therefore ideal for the analysis of non-linear time-related events in a biological system, as exemplified in the analysis of the simulated and the exemplar HgCl_2 _nephrotoxic data sets.

In comparison to PCA, the proposed method provides a more detailed picture of the time-related events including small and local changes in the time domain. In addition, the predictive properties of the proposed method can be utilised for prediction of different stages of time-dependent biological events, such as disease or a toxic perturbation studied over time. In comparison to linear multivariate regression methods (e.g. PLS and OPLS), using the time as a response variable, the piecewise multivariate approach also models non-linear time related variation. This renders a model framework describing additional time-related variation with the potential to improve prediction and interpretation if non-linear variation is dominant, in this way providing a complement to both PCA and OPLS regression against time. PCA provides an overview of the main variation in the data, while OPLS regression against time models monotonic increasing or decreasing signals over the time course. The piecewise OPLS approach provides detailed information of time related effects seen locally over the time-course as well as non-linear time-related variation.

The proposed method does not exploit autocorrelation structures in the time-series and does not providing a tool for forecasting, as do methods like Auto-Regressive Moving Average (ARMA) [40]. One reason why ARMA cannot be applied successfully to the type of data described here is the restricted number of time-points available. Non-linear modelling approaches, e.g. Artificial Neural Networks or kernel based regression methods such as Kernel PLS [41], have properties that in some cases provide models with better prediction results, however, these models are often hard or impossible to interpret in relation to the descriptor variables. For example, in the case of the mercury II chloride data set, a Kernel PLS model provided only a marginally better prediction result, with a RMSECV value of 20.0% (using a Gaussian kernel function with sigma = 0.016 and three Kernel PLS components), compared to the piecewise multivariate model. However, in many biological applications of predictive modelling it is essential to have access to transparent models that allow interpretation, rendering the proposed method beneficial compared to less transparent alternatives. Another possible limitation of the proposed method is to handle time-series samples that are severely unsynchronized, e.g. high variability in response time between animals after a perturbation.

A new perspective on the time dynamic data was achieved by placing the focus on the analysis and comparison of data based on the *changes *over the time-series, i.e. the derivative of the time dependent function. This approach provides new insights into the dynamics of the biological system, which may otherwise have been overlooked. The method could also provide the basis for fast and large-scale comparison of biological responses studied over time due to different types of perturbations. This can be accomplished by means of comparison of the piecewise weights between sub-models, which can be seen as a multivariate representation of the time-dependent events taking place in the biological system.

The common case of data sampled in a synchronized fashion has mainly been discussed in this paper. The method could easily be modified to handle cases where individuals in the training set are not sampled at the same time points, but where the sampling time is known. In this case the sub-model will be a regression model instead of a discriminant model. In such cases, the boundaries of the local time frames will be chosen so that they are sufficiently local and overlapping in the time domain. If changes between subsequent time points are very small and noisy, while the number of time-points is not limiting, the same approach can be applied by treating some neighbouring time points together in a common time-frame when estimating sub-models.

## Conclusion

Given short time-series data of high dimensionality, the proposed multivariate piecewise approach provides more detailed information compared to other commonly applied multivariate methods for analysis of post-genomic data. The temporal resolution for interpretation of the model is enhanced in the sense that it is easier to conclude which changes occur over time and when they occur, improving the interpretation of the data and providing a tool for the understanding of the biological system. The method also allows time predictions, which is an important feature in many biological and clinical applications, where time may represent e.g. disease stage and interventions are evaluated in relation to the disease stage.

## Authors' contributions

MR developed the method, evaluated it and drafted the manuscript. OC, TL and TMDE scrutinized the method. EH, JKN and JT supervised the project. All authors read and approved the final manuscript.
